# Single-Cell Transcriptomics Uncover EEF1A1-Driven Ubiquitination Dysregulation in T Cell Exhaustion and SLE Pathogenesis via STAT1-Mediated Th1/Th2 Imbalance

**DOI:** 10.1155/mi/3708640

**Published:** 2025-11-11

**Authors:** Lu Xing, Tao Wu, Hongyan Xu, Shuyun Yang, Chunyan Luan, Yinde Xu, Yi Mao, Xiaolan Li

**Affiliations:** ^1^Department of Dermatology, The Second Affiliated Hospital of Kunming Medical University, Kunming, Yunnan, China; ^2^Department of Dermatology, Kunming Children's Hospital, Children's Hospital Affiliated to Kunming Medical University, Kunming, Yunnan, China; ^3^Department of Colorectal Surgery, Third Affiliated Hospital of Kunming Medical University, Yunnan Cancer Hospital, Kunming, Yunnan, China

**Keywords:** EEF1A1, single-cell transcriptomics, STAT1, systemic lupus erythematosus, T cell

## Abstract

**Background:**

Systemic lupus erythematosus (SLE) is a complex autoimmune disorder marked by immune dysregulation and multiorgan involvement. This study investigates the role of the ubiquitination-related gene EEF1A1 in SLE pathogenesis, focusing on T cell dysfunction.

**Methods:**

Single-cell RNA sequencing (scRNA-seq) data from the GSE135779 dataset was analyzed to characterize the cellular composition of SLE samples. Clustering analysis identified 15 T cell subpopulations, with seven clusters notably depleted in SLE. Trajectory analysis indicated progressive transcriptional dysregulation during T cell differentiation. High-dimensional weighted gene coexpression network analysis (hdWGCNA) and LASSO regression highlighted EEF1A1 as a key ubiquitination-related hub gene. EEF1A1 expression was significantly elevated in SLE T cells, while its ubiquitinated form was reduced, suggesting impaired proteasomal degradation.

**Results:**

Functional assays demonstrated that EEF1A1 overexpression enhances STAT1 phosphorylation (p-STAT1) without altering total STAT1 protein levels, leading to T cell dysfunction. In vitro and in vivo experiments revealed that EEF1A1 overexpression skews the T helper 1 (Th1)/T helper 2 (Th2) balance towards a Th1-dominant phenotype. In MRL/lpr mouse models, EEF1A1 overexpression exacerbated renal pathology, including increased proteinuria and immune complex deposition.

**Conclusions:**

These findings suggest that EEF1A1 contributes to SLE pathogenesis by promoting STAT1-mediated T cell dysfunction and Th1/Th2 imbalance. EEF1A1 emerges as a potential biomarker and therapeutic target, offering new insights into the post-translational regulatory mechanisms underlying SLE.

## 1. Introduction

Systemic lupus erythematosus (SLE) is a complex autoimmune disease characterized by chronic inflammation across multiple organ systems, resulting in significant morbidity and mortality. Globally, SLE affects approximately 20–150 individuals per 100,000, with a marked female predominance, particularly among women of childbearing age [1, 2]. The clinical heterogeneity of SLE, combined with its fluctuating disease activity, presents substantial challenges for timely diagnosis and effective management. Current therapeutic strategies aim to control disease activity through the use of corticosteroids, immunosuppressive medications, and biologics [3]. However, these treatments are often limited by incomplete efficacy, adverse effects, and interpatient variability [4]. These challenges underscore the urgent need for innovative therapeutic approaches grounded in a deeper understanding of SLE pathogenesis.

Single-cell RNA sequencing (scRNA-seq) offers a comprehensive analysis of gene expression at the individual cell level, revealing cellular heterogeneity and dynamic states within the immune system [5]. In autoimmune diseases such as rheumatoid arthritis [6], multiple sclerosis [7, 8], and ankylosing spondylitis [9], scRNA-seq has uncovered cellular diversity, identified pathogenic immune subsets, and discovered potential therapeutic targets. These findings highlight the potential of scRNA-seq to provide novel insights into the pathogenesis of autoimmune diseases [10, 11]. Recent evidence suggests that disrupted ubiquitin-mediated proteostasis, a critical mechanism for maintaining cellular homeostasis through protein degradation and regulation, significantly contributes to SLE pathogenesis [12]. Specifically, dysregulation of ubiquitination-related genes in immune cells, such as T cells and monocytes, has been implicated in SLE progression, though the molecular drivers of these processes remain poorly defined. Building on these advances, we employed scRNA-seq to investigate SLE-specific mechanisms, focusing on dysregulated ubiquitination processes—post-translational modifications essential for immune regulation and protein quality control.

This study sought to integrate single-cell transcriptomics, machine learning, and advanced bioinformatics analyses to systematically investigate the cellular, molecular, and functional characteristics of SLE. Through integrative analysis of clinical cohorts and preclinical models, we identified EEF1A1 as a key regulator of T cell dysfunction and systemic inflammation. By profiling immune cell types and gene expression patterns, we further explored the mechanistic interplay between transcription factor (TF) activation and T helper 1 (Th1)/T helper 2 (Th2) immune imbalance, aimed to uncover novel insights into SLE pathogenesis and identify potential therapeutic targets.

## 2. Materials and Methods

### 2.1. Study Design

This study was approved by the Ethics Committee of the Second Affiliated Hospital of Kunming Medical University and conducted in accordance with the principles outlined in the Declaration of Helsinki. Written informed consent was obtained from all participants. The study included eight patients with SLE who met the European League Against Rheumatism (EULAR)/American College of Rheumatology (ACR) classification criteria [13] and had a SLE disease activity index (SLEDAI) score of ≥4, as well as six sex-matched healthy controls (Table S1). The tissue samples from these participants were utilized for experimental validation assays. Given the exploratory nature of this study, the sample size of eight SLE patients and six healthy controls was appropriate for preliminary discovery and hypothesis generation.

### 2.2. Cell Type Annotation

Cell type annotation was performed using the SingleR package (v1.4.1). Differentially expressed genes (DEGs) or highly variable genes (HVGs) associated with known cell types were identified via differential expression analysis or ANOVA. For each single cell, Spearman's rank correlation coefficients were calculated between its expression profile and reference samples in the dataset. The 80th percentile of correlation coefficients across reference samples within the same cell type was used as the annotation score. To ensure robustness, reference cell types with maximum score differences ≤ 0.05 from the highest score were retained as the final annotated cell types.

### 2.3. Single-Cell Progression Analysis

The single-cell transcriptomic data analyzed in this study were sourced from the publicly available dataset GSE135779. Single-cell transcriptomic data were processed using the Seurat package (v4.1.0) [14, 15]. Cells expressing 200–2500 RNA features were included, and mitochondrial RNA content was standardized at a 10% threshold. Batch effects were removed using the Harmony algorithm. The data were scaled and subjected to principal component (PC) analysis (PCA) with the “ScaleData” and “RunPCA” functions, respectively, using 10 PCs for clustering. Clusters were visualized via *t*-SNE plots, and unsupervised clustering was performed using the Leiden algorithm (resolution = 0.5). Cell-type annotation was automated using the SingleR package based on marker genes identified in each cluster. Subsequently, T cells and monocytes were selected for further PCA to identify subtypes within these populations.

### 2.4. Bulk Transcriptome Preprocessing

Publicly available datasets from the Gene Expression Omnibus (GEO) database were analyzed to compare SLE patients and healthy controls. The datasets included GSE81622 (Illumina HT microarray) and GSE50772 (Affymetrix microarray). To harmonize cross-platform data, batch effects were removed using the ComBat algorithm (R package sva). Transcript expression levels were normalized using log_2_ (transcripts per million [TPM] + 1) transformation, where TPM values were calculated to standardize gene expression across samples.

### 2.5. Machine Learning Model Construction

To evaluate the diagnostic potential of feature genes identified using the high-dimensional weighted gene coexpression network analysis (hdWGCNA) R package, we implemented a comprehensive machine learning framework. The predictive performance of these genes was assessed through receiver operating characteristic (ROC) analysis on datasets GSE81622 and GSE50772, with the area under the curve (AUC) serving as the primary evaluation metric. For gene screening, we employed the “randomForest,” “e1071,” and “glmnet” packages, utilizing the random forest, SVM-RFE, and LASSO algorithms, respectively. Specifically, random forest selected variables based on a MeanDecreaseGini value greater than zero; SVM-RFE determined variables by identifying the *λ* that minimized the classification error rate; and LASSO extracted genes corresponding to the minimum *λ* value with nonzero coefficients.

To optimize feature selection, we integrated 13 distinct machine learning algorithms and systematically tested 101 algorithm combinations. These included: neural network (multilayer perceptron [MLP]), logistic regression, linear discriminant analysis (LDA), quadratic discriminant analysis (QDA), *k*-nearest neighbor (*k*-NN), decision tree, random forest, XGBoost ridge regression, elastic net regression, lasso regression, support vector machine, gradient boosting machine, stepwise logistic regression, and naive Bayesian algorithm [16].

### 2.6. Gene Set Variation Analysis (GSVA) Enrichment Analysis

Differential gene expression between SLE and control samples was analyzed using the “limma” package in R. Functional enrichment analysis of feature genes was performed using the “ClusterProfiler” package (v4.0). The GSVA algorithm was applied to assess alterations in the activities of Kyoto Encyclopedia of Genes and Genomes (KEGG) pathways associated with the selected feature genes [17]. A *p*-value threshold of <0.05 was used to determine statistical significance.

### 2.7. Pseudotime Analysis

The Monocle2 algorithm (v2.22.0) was employed to infer developmental and differentiation trajectories of cells and to elucidate the relationships between various cell states. Trajectory analysis was conducted using Monocle software (v2.18.0) to investigate the differentiation processes of T cells and monocytes. Additionally, the single-cell regulatory network inference and clustering ([SCENIC] v1.2.4) tool was utilized to uncover regulatory interactions between TFs and their target gene [18]. Significantly expressed regulatory factors were identified using the “limma” package, with a *p*-value threshold of <0.05 for statistical significance.

### 2.8. Cellular Communication Analysis

The “CellChat” package was used to analyze cell interactions and communication. This tool enabled the deduction of significant biological interactions between cells and the computation of interaction probabilities and significance levels [19]. The relationships and significance between cells were visualized using chord diagrams and bubble plots.

### 2.9. Single-Cell Population Heterogeneity Analysis

Following cell type annotation, we systematically quantified the activity of ubiquitination-related gene families across all identified cell types using five complementary scoring algorithms: AUCell, UCell, singscore, ssGSEA, and the AddModuleScore function. To validate specificity, background gene sets were randomly selected from the scoring gene pools. The mean expression levels of these background genes were then compared against those of the ubiquitination-focused gene sets to rigorously assess pathway-specific enrichment patterns.

### 2.10. Cell Culture

Frozen cells were thawed in a 37°C water bath, sterilized with 75% ethanol, then centrifuged (300 × *g*, 3 min). Pellets were resuspended in DMEM (10% FBS, 1% penicillin/streptomycin) and seeded into T25 flasks. Cultures were maintained at 37°C, 5% CO_2_. When cells reached 80% confluency, cells were detached with 0.25% trypsin–EDTA, neutralized with medium, and either reseeded or frozen in cryoprotective medium using a controlled-rate freezer to −80°C overnight before long-term storage in liquid nitrogen.

### 2.11. Flow Cytometry

Cells were pelleted (300 g, 5 min), washed twice with PBS, and resuspended in 50 µL PBS. For surface staining, 5 µL anti-CD4 (Elabscience, E-AB-F1097C) was added and incubated in the dark at 4°C for 30 min. Cells were then fixed and permeabilized using Cyto-Fast Fix/Perm buffer for 20 min at 4°C, followed by two PBS washes. Intracellular cytokines were stained by adding 5 µL each of anti-IL-2 (Elabscience, E-AB-F1201E) and anti-IL-4 (Elabscience, E-AB-F1204D), with a 30 min incubation at 4°C in the dark. Stained cells were resuspended in 400 µL PBS and filtered through nylon mesh. Data were acquired on a BD FACSCalibur (10,000 events/sample) and analyzed using FlowJo v10.0. Statistical analysis was performed in GraphPad Prism.

### 2.12. Animal Experiments

Female MRL/lpr mice (8 weeks old, *n* = 10) and C57BL/6 mice (8 weeks old, *n* = 5) were purchased from Sibeifu (Beijing) Biotechnology Co., Ltd. and Sikebeisi (Henan) Biotechnology Co., Ltd., respectively. All mice were maintained under SPF conditions (12 h light/dark cycle, 22 ± 2°C, 35%–60% humidity) with ad libitum access to food and water. After a two-day acclimation (day 0), MRL/lpr mice were randomized into two groups (*n* = 5 each) and received a single tail-vein injection at week 4 of either empty vector (OE-NC) or eEF1A1-expressing plasmid (OE-eEF1A1). Age-matched C57BL/6 mice served as healthy controls and received saline. Urinary protein (UP) was measured at weeks 4, 8, and 12. At week 12, mice were euthanized by pentobarbital sodium overdose (100 mg/kg). Spleens, kidneys, and blood were collected, snap-frozen in liquid nitrogen, and stored at −80°C for downstream analyses.

### 2.13. Immunofluorescence (IF)

Paraffin-embedded kidney sections (5 µm) were deparaffinized, rehydrated, and subjected to microwave antigen retrieval in citrate buffer (pH 6.0). After blocking in 5% BSA for 30 min, sections were incubated overnight at 4°C with mouse anti-C3 (Abcam ab11862, 1:100) and mouse anti-immunoglobin G (IgG) (Santa Cruz sc-2025, 1:100). Following three PBS washes, secondary antibodies (goat antimouse IgG H and L [Alexa Fluor 555, red fluorescence] for C3; goat antimouse IgG H and L [Alexa Fluor 488, green fluorescence] for IgG) were added and incubated at 37°C for 20 min. Nuclei were stained with DAPI (excitation: 330–380 nm, emission: 420 nm, blue fluorescence) for 5 min, then slides were mounted in antifade medium and imaged on a fluorescence microscope (Nikon, TS2-S-SM).

### 2.14. RNA Isolation and Quantitative PCR (qPCR)

Total RNA was extracted with TRIzol reagent (Thermo Fisher Scientific, 15596-018CN) and quantified by NanoDrop (*A*_260_/*A*_280_ ≥ 1.8). First-strand cDNA was synthesized using the SureScript kit (Servicebio) under the following conditions: 25°C for 5 min, 50°C for 15 min, and 85°C for 5 s, then held at 4°C. For qPCR, cDNA was diluted 1:5 and amplified with BlazeTaq qPCR Mix (Servicebio) and gene-specific primers (Table 1). The program was: 95°C for 1 min, then 40 cycles of 95°C for 20 s, 55°C for 20 s, and 72°C for 30 s. Ct values were used to calculate relative expression by the 2^–ΔΔCt^ method.

### 2.15. Western Blot Analysis

Cells were washed in ice-cold PBS, lysed in RIPA buffer (Servicebio, G2002-30 mL) with protease inhibitors (Proteintech, PR20032), and centrifuged at 16,000 × *g* (4°C, 15 min). Protein concentration was determined by BCA assay (Beyotime, P0009). Samples were mixed with loading buffer, boiled (100°C, 10 min), then resolved by SDS-PAGE (Solarbio, S1010), and transferred to PVDF membranes. After blocking in 5% BSA (Solarbio, CR2302110) (1 h, RT), membranes were incubated overnight at 4°C with primary antibodies against eEF1A1 (Abcam, ab157455, 1:10000), STAT1 phosphorylation (p-STAT1) (Affinity, AF3300, 1:2000), STAT1 (Affinity, AF6300, 1:2000), and β-actin (Proteintech, 66009-1-Ig, 1:25000), followed by HRP-conjugated secondary antibodies (goat antirabbit IgG [Servicebio, GB23303, 1:3000] or goat antimouse IgG [Servicebio, GB23301, 1:5000]) (40 min, RT). Bands were visualized by ECL (Affinity, KF8001), imaged chemiluminescently, quantified in ImageJ, and analyzed in GraphPad Prism.

### 2.16. Enzyme-Linked Immunosorbent Assay (ELISA)

UP levels were measured using a mouse UP ELISA kit (Meimian, MM-44287M2). After equilibrating the plate 60 min at room temperature, 50 µL of standards or samples (blanks: buffer only) were added to wells. We then added 100 µL HRP antibody, incubated at 37°C for 60 min, and washed wells five times. Next, 50 µL substrate was incubated in the dark at 37°C for 15 min, followed by 50 µL stop solution. Absorbance at 450 nm was read within 15 min.

### 2.17. Statistical Analysis

Bioinformatic workflows and network plots were created in R and Cytoscape. Experimental data are presented as mean ± SEM and were analyzed in GraphPad Prism 9.0. Two-group comparisons used unpaired two-tailed *t*-tests; multigroup comparisons used one-way ANOVA with post hoc tests. Statistical significance was defined as *p*-values < 0.05 (*⁣*^*∗*^), *p* < 0.01 (*⁣*^*∗∗*^), *p* < 0.001 (*⁣*^*∗∗∗*^), and *p* < 0.0001 (*⁣*^*∗∗∗∗*^) are indicated as significant.

## 3. Results

### 3.1. Single-Cell Transcriptomic Analysis Reveals Ubiquitination Pathway Dysregulation in SLE Immune Cell Subsets

**Figure 1:**
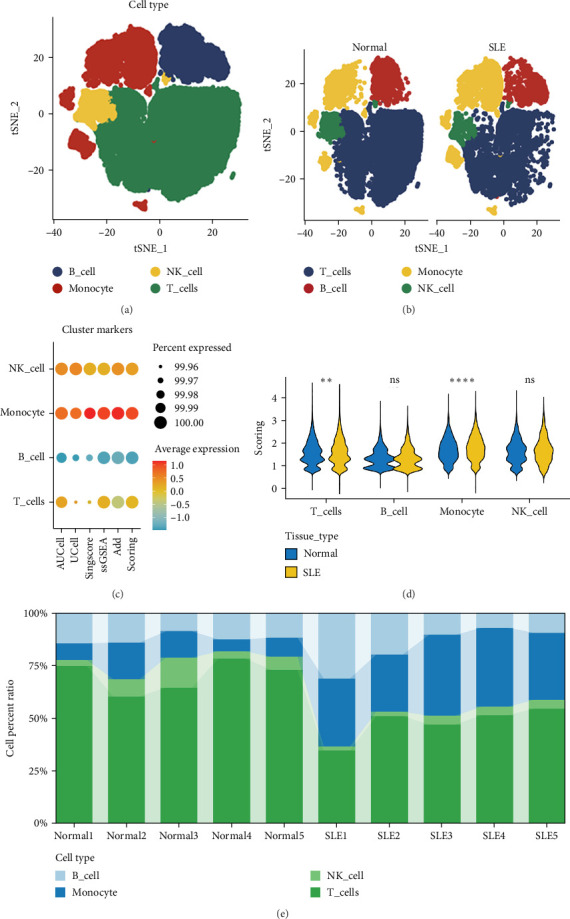
Single-cell transcriptomic profiling of SLE immune subsets. (a) tSNE visualization of immune cell clusters generated using Seurat. (b) Proportional changes in major immune cell types between SLE and controls. (c) Computational assessment of ubiquitination-related pathway activity across immune cell types. (d) Differential expression analysis of ubiquitination-related genes in T cells and monocytes. (e) Quantification of immune cell proportions across specimens. ns *p* ≥ 0.05; *⁣*^*∗*^*p* < 0.05; *⁣*^*∗∗*^*p* < 0.01; *⁣*^*∗∗∗*^*p* < 0.001; *⁣*^*∗∗∗∗*^*p* < 0.0001.

To investigate the heterogeneity of the SLE microenvironment influenced by ubiquitination-related gene families, we analyzed scRNA-seq data from SLE patients and healthy controls, utilizing the GSE135779 dataset. Dimensionality reduction and clustering analyses were performed using the Seurat pipeline, followed by cell type annotation with the SingleR package. This approach delineated four principal immune cell populations, specifically T cells, natural killer (NK) cells, monocytes, and B cells (Figure 1A). Subsequent analysis of cell type proportions revealed a notable decrease in T cells and an expansion of monocytes in SLE samples compared to healthy controls (Figure 1B).

To systematically profile ubiquitination-related gene family expression across immune cell subsets, we first curated a pathway-defined gene set from the Molecular Signatures Database (MSigDB), then implemented five computational algorithms (AUCell, UCell, singscore, ssGSEA, and AddModuleScore) for comprehensive pathway activity quantification. These analyses indicated significant activation of ubiquitination pathways in all cell types except B cells (Figure 1C). Differential expression analysis between SLE patients and healthy controls highlighted substantial alterations in ubiquitination-related gene expression within T cell (*p* < 0.01) and monocyte subsets (*p* < 0.0001) (Figure 1D). Cellular proportion quantification revealed pronounced alterations in immune cell distributions across specimens, providing direct evidence for disease-associated restructuring of the SLE microenvironment (Figure 1E).

### 3.2. Subset-Specific Analysis Reveals T Cell Dysfunction in SLE

**Figure 2:**
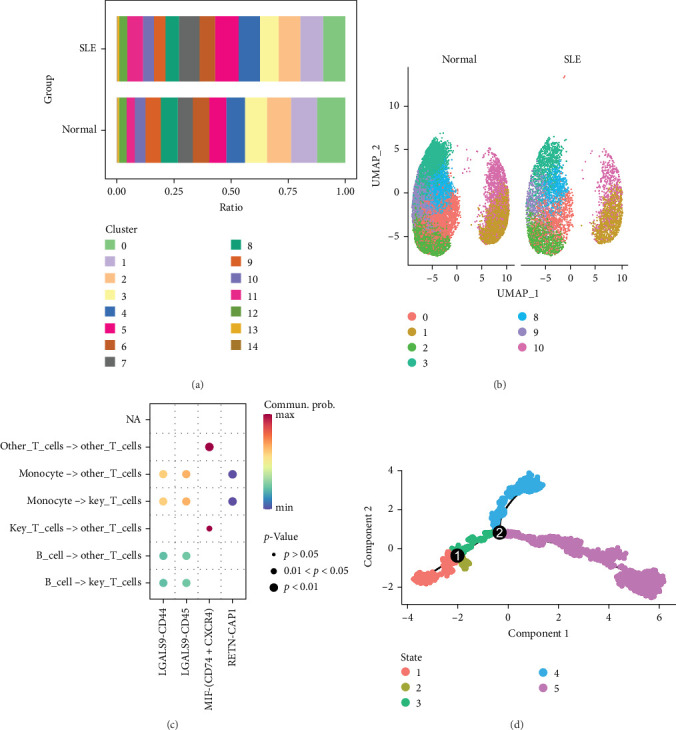
T cell subset dynamics in SLE. (a) Bar plot indicating the proportion of T cell clusters between SLE and controls. (b) UMAP plot showing the distribution of key T cell subpopulations. (c) Bubbleplot showing the significant ligand–receptor pairs between cells. (d) Pseudotime trajectory analysis of T cell differentiation using Monocle.

To investigate the pathogenic contributions of T cell subsets in SLE, we conducted subset-specific analyses. Clustering of T cells identified 15 subclusters, among which seven (clusters 0, 1, 2, 3, 8, 9, and 10) were significantly depleted in SLE patients compared to healthy controls. Given the overall reduction of T cells in SLE, these diminished subclusters—designated as key T cell clusters—were selected for detailed characterization (Figure 2A, B).

Ligand–receptor interaction analysis revealed robust crosstalk between key T cell clusters and monocytes via LGALS9-CD45 signaling (Figure 2C). Pseudotime trajectory analysis demonstrated that key T cell clusters exhibited progressive functional impairment from early to late differentiation stages, implicating T cell dysfunction as a hallmark of SLE progression (Figure 2D).

### 3.3. Identifying Key Modules in SLE-Associated T Cell Subclusters by hdWGCNA

**Figure 3:**
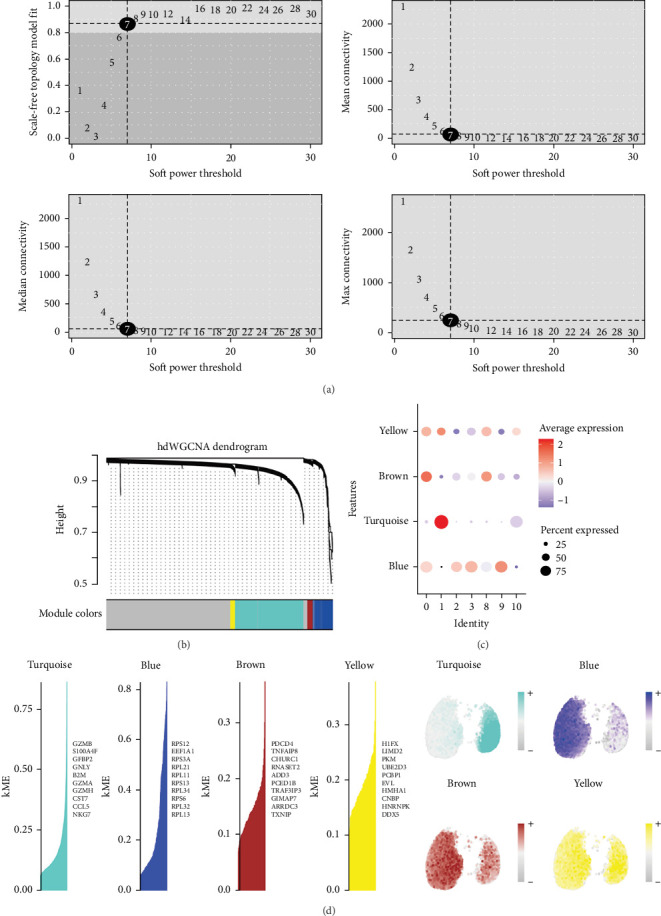
Identifying key modules in SLE-associated T cell subclusters by hdWGCNA. (a) Soft-thresholding power selection for hdWGCNA. (b) Cluster dendrogram of gene coexpression modules. (c) Module eigengene-based connectivity (KME) analysis. (d) Spatial expression patterns of gene modules across T cell subclusters.

To elucidate the molecular underpinnings of T cell dysfunction in SLE, we performed hdWGCNA on T cell subclusters (0, 1, 2, 3, 8, 9, and 10). A soft-thresholding power of 7 was selected to construct a scale-free network, resulting in the identification of four distinct gene modules: yellow, brown, blue, and turquoise (Figure 3).

We calculated the module eigengene-based connectivity (KME) for each gene to assess its correlation with the respective module eigengene. All four modules exhibited elevated KME values, indicating strong associations between hub genes and their corresponding modules (Figure 3C). Spatial expression visualization confirmed robust and compartmentalized activity patterns of these modules across T cell subpopulations (Figure 3D). Notably, SLE-associated T cell subclusters demonstrated marked transcriptional enrichment of all four modules, underscoring their collective contribution to T cell dysfunction in SLE pathogenesis.

### 3.4. Identification and Clinical Validation of the Ubiquitination-Related Driver Gene EEF1A1 in SLE T Cells

**Figure 4:**
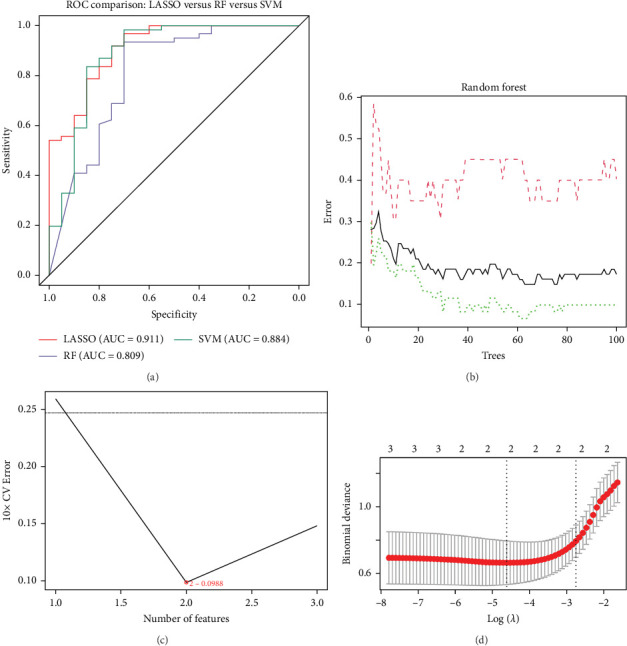
Evaluation of machine learning models for hub gene screening. (a) ROC curve analysis of three models. (b) Error rate trajectory of the random forest model. (c) Feature selection error in SVM-RFE. (d) Tuning parameter selection in LASSO regression.

To validate the biological significance of the hub genes identified from hdWGCNA modules, we integrated transcriptomic profiles from two independent SLE cohorts (GSE81622 and GSE50772). After correcting for batch effects, we applied machine learning models including random forest, SVM-RFE, and LASSO regression. ROC curve evaluation showed that all three models achieved AUC values above 0.8, with LASSO exhibiting the highest AUC of 0.911 (Figure 4A). The error rate of random forest stabilized after 70 trees (Figure 4B), the minimum error rate for SVM-RFE was 0.0988 (Figure 4C), and the optimal *λ* value for LASSO was 0.009887539 (Figure 4D). Based on these results, LASSO regression was selected as the preferred model due to its superior performance.

**Figure 5:**
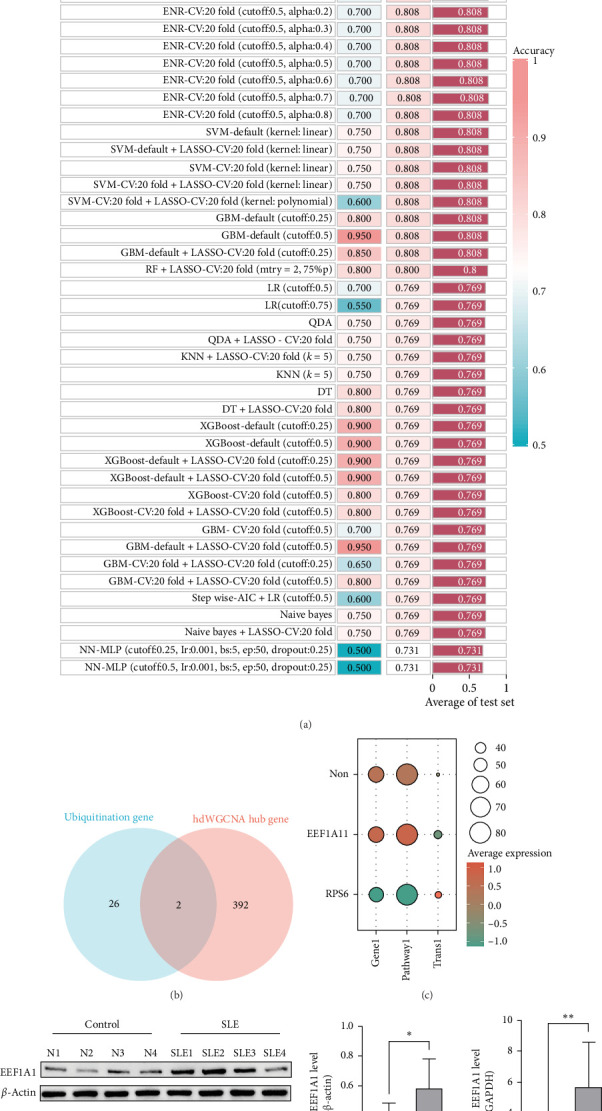
Screening and validation of the ubiquitination-related driver gene EEF1A1. (a) Multialgorithm consistency evaluation of candidate genes. (b) Overlap analysis with ubiquitination-related gene sets. (c) Expression-based filtering in T cell subclusters. (d–f) Validation of expression and ubiquitination levels in clinical samples. *⁣*^*∗*^*p* < 0.05, *⁣*^*∗∗*^*p* < 0.01, *⁣*^*∗∗∗*^*p* < 0.001, *⁣*^*∗∗∗∗*^*p* < 0.0001.

Using LASSO, we identified six key pathogenic genes: RPS5, EEF1A1, TCP1, TCEAL8, TCAF2, and PRS6. These candidates were further evaluated using 13 machine learning algorithms in 101 combinatorial approaches, and multialgorithm cross-validation demonstrated consistent diagnostic performance (Figure 5A).

From the 394 hub genes identified across four modules, we prioritized ubiquitination-related candidates through MSigDB screening. Overlap analysis with 28 curated ubiquitination genes revealed EEF1A1 and RPS6 as key genes (Figure 5B). Subsequent filtering based on expression activity in ubiquitination-enriched T cell subclusters excluded RPS6 due to low activity scores (*p* < 0.05, logFC > 1) (Figure 5C), thereby nominating EEF1A1 as the primary ubiquitination-associated driver gene involved in SLE T cell dysfunction.

To further validate these findings in clinical samples, we analyzed peripheral blood from eight SLE patients and six matched healthy controls. As shown in Figure 5D–F and Figure S1A,B, EEF1A1 was significantly upregulated in SLE, whereas its ubiquitinated form was markedly reduced, suggesting reduced proteasomal degradation capacity. These results support the pathological relevance of EEF1A1 in SLE and establish it as a potential biomarker.

### 3.5. EEF1A1 Amplifies SLE Progression Through Multifaceted Pathway Dysregulation and STAT1-Mediated T Cell Dysfunction

**Figure 6:**
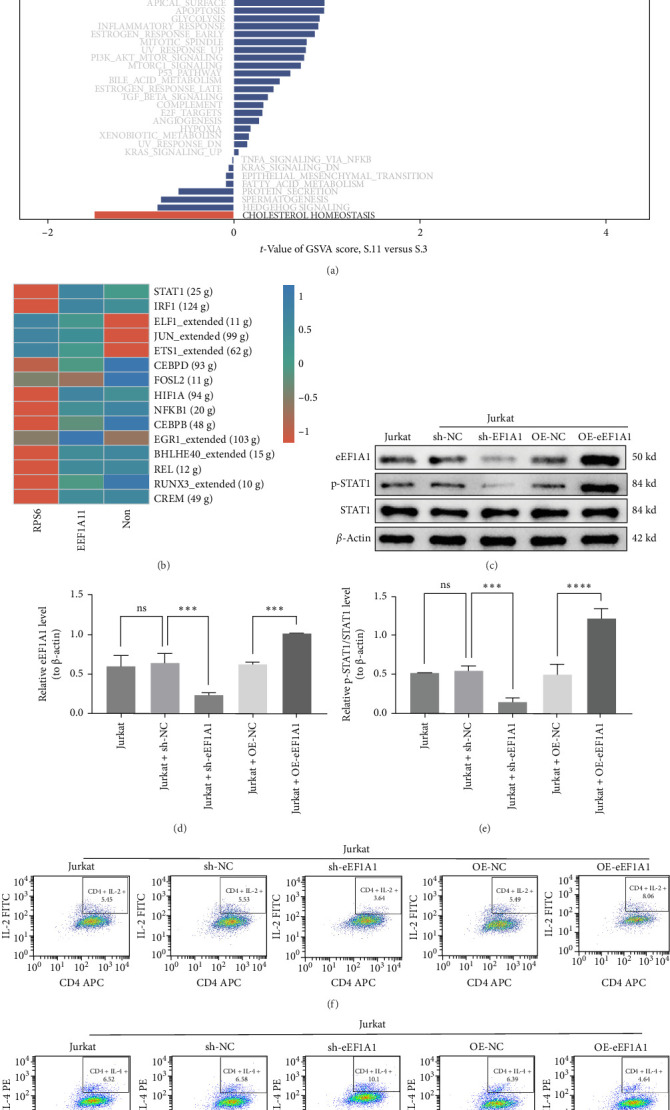
Functional impact of EEF1A1 on T cell signaling and differentiation in SLE. (a) Pathway enrichment analysis using GSVA. (b) Transcription factor regulatory network from SCENIC. (c–e) Effects of EEF1A1 manipulation on STAT1 phosphorylation. (f, g) Th1/Th2 ratio analysis by flow cytometry. ns *p* ≥ 0.05; *⁣*^*∗*^*p* < 0.05; *⁣*^*∗∗*^*p* < 0.01; *⁣*^*∗∗∗*^*p* < 0.001; *⁣*^*∗∗∗∗*^*p*  < 0.0001.

GSVA showed that EEF1A1 overexpression disrupts multiple pathways critical to T cell function, including abnormal activation of NOTCH and WNT signaling, enhanced interferon and IL-6 responses, DNA repair defects, and impaired oxidative phosphorylation (Figure 6A). These disruptions collectively suggest that EEF1A1 undermines T cell homeostasis and promotes SLE pathology. SCENIC analysis identified 15 dysregulated TFs in SLE, which indicated that STAT1 transcriptional activation is mechanistically linked to EEF1A1 upregulation (Figure 6B).

Our study demonstrated a strong correlation between EEF1A1 expression and STAT1 signaling activation. To investigate the functional relationship between EEF1A1 and STAT1 signaling, we constructed EEF1A1-overexpressing and silencing plasmids for Jurkat T cell transfection (Figure S1C, D). In Jurkat T cells, EEF1A1 knockdown significantly suppressed p-STAT1 without altering total STAT1 protein levels, whereas EEF1A1 overexpression enhanced p-STAT1 levels (*p* < 0.001; Figure 6). Reverse transcription qPCR (RT-qPCR) confirmed that EEF1A1 manipulation did not affect STAT1 mRNA levels (Figure S2A, B), indicating that EEF1A1 regulates STAT1 activation at the post-translational level.

Accumulating evidence suggests that imbalances in Th1 and Th2 cell populations contribute to SLE pathogenesis. Flow cytometry analysis showed that EEF1A1 knockdown reduced Th1 cell populations and decreased the Th1/Th2 ratio, whereas EEF1A1 overexpression promoted Th1 differentiation and increased the Th1/Th2 ratio (*p* < 0.001; Figure 6 and Figure S2C, D). These findings establish the EEF1A1-STAT1 signaling axis as a central driver of T cell dysfunction in SLE.

To further investigate, qPCR was performed to examine the expression of key cytokines (IFN-γ, IL-4, and IL-17) and TFs (T-bet, GATA3) associated with Th1, Th2, and Th17 cell differentiation. The results showed that silencing eEF1A1 significantly suppressed the expression of T-bet, IFN-γ, and IL-17, whereas overexpression of EEF1A1 enhanced their expression (*p* < 0.05; Figure S3A–C). Conversely, eEF1A1 silencing increased the expression of GATA3 and IL-4, while EEF1A1 overexpression reduced their levels (*p* < 0.05; Figure S3D, E). These findings indicate that eEF1A1 may promote Th1 differentiation and IFN-γ secretion by upregulating T-bet, as well as facilitate Th17 activation and IL-17 secretion, while inhibiting Th2 differentiation and IL-4 secretion through downregulation of GATA3.

### 3.6. EEF1A1 Overexpression Exacerbates Renal Injury in SLE Mice

**Figure 7:**
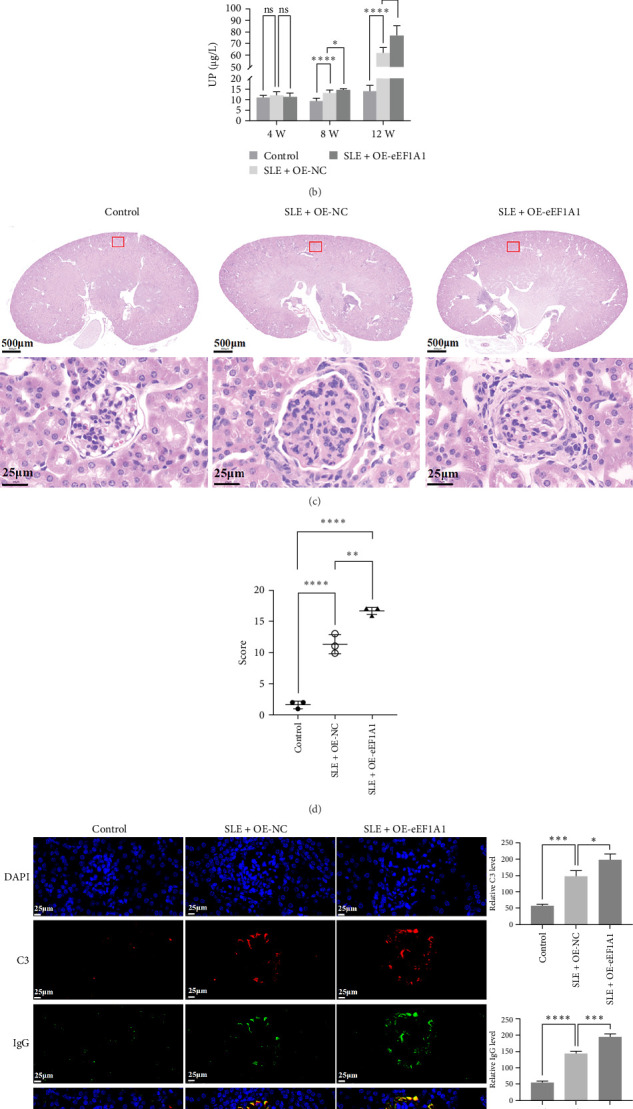
In vivo assessment of EEF1A1-driven renal injury in SLE mice. (a) Experimental timeline for SLE mouse modeling. (b) Urinary protein quantification via ELISA. (c) Renal histopathology (H and E staining). (d) Histological scoring of kidney damage. (e) Immunofluorescence detection of C3 and IgG deposition. ns *p* ≥ 0.05; *⁣*^*∗*^*p* < 0.05; *⁣*^*∗∗*^*p* < 0.01; *⁣*^*∗∗∗*^*p* < 0.001; *⁣*^*∗∗∗∗*^*p* < 0.0001.

To investigate the role of eEF1A1 in SLE, we utilized MRL/lpr mice as an SLE model and C57BL/6 mice as healthy controls. At week 4 of modeling, MRL/lpr mice received tail vein injections of either an OE-NC or an eEF1A1-overexpressing plasmid (OE-eEF1A1). Renal function and pathology were assessed at week 12 (Figure 7A).

UP levels, measured by ELISA, showed no significant differences among groups at week 4. However, by week 8, the SLE model group exhibited elevated proteinuria compared to controls, indicating impaired renal filtration. This effect was more pronounced in the OE-eEF1A1 group. By week 12, proteinuria levels further increased in the SLE model group and were significantly higher in the OE-eEF1A1 group, suggesting that eEF1A1 overexpression exacerbates renal dysfunction (Figure 7B).

Histopathological analysis using hematoxylin and eosin (H and E) staining revealed classic SLE-associated renal lesions in SLE model mice, including glomerular architectural disruption, mesangial matrix expansion, and perivascular leukocyte infiltration. These pathlogical changes were more severe in the OE-eEF1A1 group (Figure 7C). Quantitative histological scoring demonstrated a significant exacerbation of renal pathology in OE-eEF1A1 mice, with composite scores increasing from 11.3  ± 1.53 in OE-NC mice to 16.7 ± 0.58 in OE-eEF1A1 mice, with significant differences (Figure 7D and Table S2).

IF analysis showed markedly increased deposition of complement component C3 and IgG in the glomeruli of SLE model mice compared to controls. This deposition was further enhanced in the OE-eEF1A1 group, indicating that eEF1A1 overexpression may promote immune complex-mediated nephropathy (Figure 7E).

These findings indicate that eEF1A1 overexpression in SLE model mice leads to heightened immune complex deposition and worsened renal pathology, underscoring its potential role in exacerbating SLE-associated kidney damage.

### 3.7. EEF1A1 Enhances p-STAT1 and Disrupts Th1/Th2 Balance In Vivo

**Figure 8:**
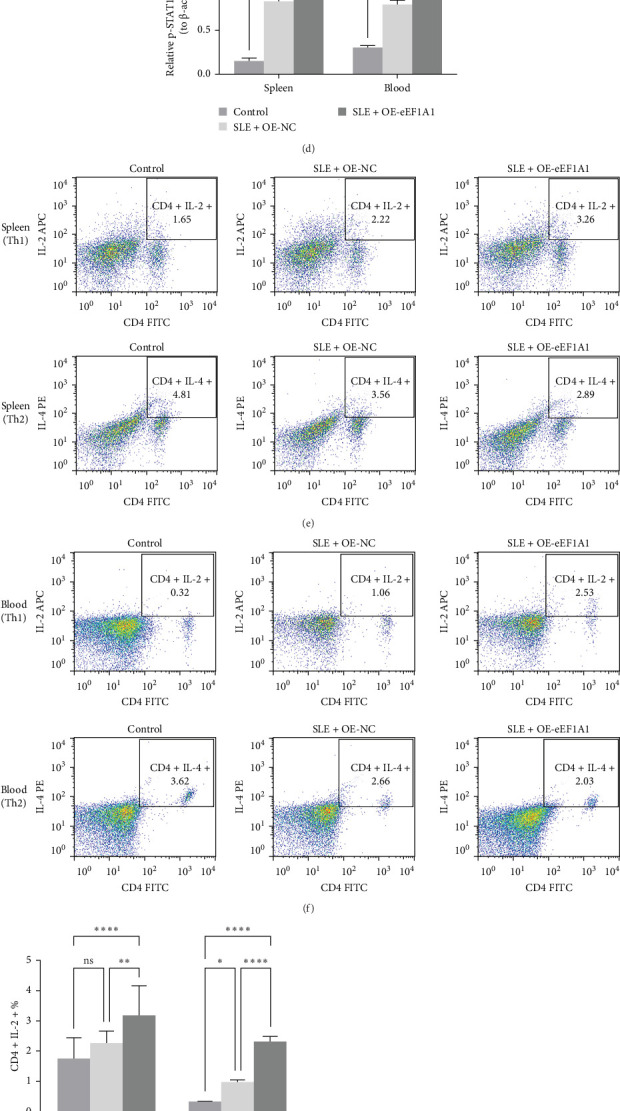
EEF1A1-mediated STAT1 activation and Th1/Th2 imbalance in SLE mice. (a–d) Western blot analysis of EEF1A1 and p-STAT1 in spleen and peripheral blood. (e, f) Flow cytometry quantification of Th1/Th2 subsets. (g, h) Th1/Th2 ratio analysis. ns *p* ≥ 0.05; *⁣*^*∗*^*p* < 0.05; *⁣*^*∗∗*^*p* < 0.01; *⁣*^*∗∗∗*^*p* < 0.001; *⁣*^*∗∗∗∗*^*p* < 0.0001.

To investigate the role of eEF1A1 in SLE, we analyzed its expression in spleen and peripheral blood samples from SLE model mice and healthy controls. Western blot analysis revealed significantly elevated eEF1A1 levels in the SLE group (Figure 8A,B), suggesting its potential involvement in disease pathogenesis. Further, overexpression of eEF1A1 in SLE model mice led to a notable increase in p-STAT1 levels, while total STAT1 protein remained unchanged (Figure 8C,D). This indicates that eEF1A1 may enhance STAT1 activation through post-translational modification, aligning with our in vitro findings.

Flow cytometry was employed to assess the impact of eEF1A1 on T-helper cell subsets. Compared to healthy controls, SLE model mice exhibited an increased proportion of Th1 cells and a decreased proportion of Th2 cells in both spleen (Figure 8E) and peripheral blood (Figure 8F), indicating a Th1/Th2 imbalance associated with SLE. Notably, eEF1A1 overexpression further amplified the Th1 cell population without significantly affecting Th2 cells, exacerbating this imbalance.

Analysis of the Th1/Th2 ratio corroborated these findings, with SLE model mice showing a higher ratio than controls, and eEF1A1 overexpression leading to a further increase (Figure 8G,H).

Collectively, these results suggest that eEF1A1 contributes to SLE pathogenesis by promoting p-STAT1 and disrupting the Th1/Th2 balance.

## 4. Discussion

Our investigation demonstrates that EEF1A1, a ubiquitination-related gene, drives SLE pathogenesis through integrated mechanisms. Specifically, single-cell transcriptomics uncovered activation of the ubiquitination pathway in SLE T cells and monocytes, where EEF1A1 was identified as a hub gene associated with T cell dysfunction. While monocytes exhibited elevated proinflammatory signaling and antigen-presenting potential, T cells showed prominent signatures of dysfunction, including enhanced ubiquitination activity and altered cytokine profiles. Although this study also observed monocyte expansion and ubiquitination pathway activation in SLE patients, subsequent functional analyses focused primarily on T cells, based on the following considerations. Substantial existing evidence has firmly established that T cell dysfunction is a central driver of autoimmune damage in SLE [20, 21]. Inflammatory factors such as monocyte-derived IFN-α/γ and IL-6 can further lower the activation threshold of T cells and induce T cell subset imbalance, thereby forming a “monocyte-T cell” positive feedback loop [22]. We thus propose that monocyte abnormalities may induce and sustain T cell dysfunction through the persistent release of proinflammatory mediators, which led us to prioritize T cell dysfunction as the core focus of functional investigation in this study.

Given the central role of T cells in maintaining immune tolerance and orchestrating adaptive responses [20–23], we focused our experimental validation on T cell subtypes—particularly the Th1/Th2 axis—known to be disrupted in SLE. Subsequently, we found that EEF1A1 overexpression enhances p-STAT1, thereby promoting proinflammatory Th1 differentiation and disrupting Th1/Th2 balance.

In vivo studies further revealed that EEF1A1 overexpression in SLE model mice amplifies p-STAT1 and Th1 skewing in both spleen and peripheral blood, directly linking its molecular effects to systemic immune dysregulation. Importantly, in vivo validation using MRL/lpr mice further demonstrated that EEF1A1 exacerbates renal injury both by increasing immune complex deposition and aggravating glomerular damage, thereby linking its dual pathogenic effects to clinical disease manifestations. Notably, the pathogenic role of EEF1A1 is not restricted to renal tissue. In the skin, upregulation of EEF1A1 in keratinocytes exacerbates viral and psoriatic inflammation by suppressing antiviral innate immune responses [24]. In the joints, synovial fibroblasts utilize EEF1A1 to accelerate the translation of IL-6 and IL-8, establishing a positive feedback loop of “translation-inflammation coupling” that promotes the progression of rheumatoid arthritis [25]. This divergence in expression levels and functional orientation across organs suggests that tissue-specific regulation of EEF1A1 may be a key determinant of organ involvement patterns in autoimmune diseases, a hypothesis that warrants further validation using skin- or joint-specific experimental models.

These findings position EEF1A1 as a central regulator of SLE progression, bridging ubiquitination defects, immune dysregulation, and end-organ damage. Although EEF1A1 is a ubiquitously expressed “housekeeping” protein, single-cell atlas data reveal its significant upregulation specifically in lupus renal monocytes and T cells, suggesting a disease-specific window of action [26]. CRISPR-based functional mapping further identified that its 219–247 amino acid segment specifically drives autoantibody production [27]. Importantly, CD11c-targeted lipid nanoparticles delivering EEF1A1-siRNA significantly reduced autoantibody levels in MRL/lpr mice without affecting global protein synthesis [28]. To circumvent potential off-target effects resulting from its essential housekeeping functions, future efforts should prioritize the development of T cell-targeted nanocarriers or allosteric inhibitors to achieve precise therapeutic intervention. Ubiquitination is critical for maintaining immune homeostasis by regulating protein degradation and signaling [29, 30].

Our scRNA-seq analysis revealed broad activation of ubiquitination pathways in SLE T cells and monocytes, suggesting a compensatory response to proteasomal dysfunction. However, reduced ubiquitinated EEF1A1 in SLE patient serum implies impaired degradation of this protein, leading to its pathological accumulation. This aligns with prior studies linking ubiquitination defects to autoimmune diseases [31, 32], but our work uniquely identifies EEF1A1 as a SLE-specific driver.

The hdWGCNA-derived gene modules further highlight its role in coordinating transcriptional programs that destabilize T cell function. Functional enrichment of the four modules (yellow, brown, blue, and turquoise) using KEGG pathways revealed associations with NF-κB signaling (brown module), oxidative phosphorylation (blue), and interferon response (turquoise), collectively mirroring SLE-associated pathways.

Notably, in vivo validation confirmed that EEF1A1 overexpression drives Th1/Th2 imbalance—a hallmark of SLE—by enhancing STAT1 activation, underscoring its systemic impact beyond transcriptional regulation. Our study revealed a strong correlation between EEF1A1 expression and STAT1 signaling activation, where p-STAT1 drives Th1 polarization in multiple disease contexts [33, 34]. STAT1 activation is a hallmark of SLE, driving interferon signatures and Th1 polarization [35, 36]. We demonstrate that EEF1A1 enhances p-STAT1 without altering total STAT1 levels, suggesting post-translational regulation. This mechanism is conserved across species, as EEF1A1-overexpressing mice exhibited elevated p-STAT1 and Th1 skewing in both spleen and peripheral blood, mirroring human SLE profiles. Notably, STAT1 activation by EEF1A1 may synergize with other dysregulated pathways to amplify inflammation [37]. Our findings differ from most previous studies focusing on STAT3/Th17 imbalance in SLE [38, 39], proposing EEF1A1-STAT1 as a novel axis for therapeutic intervention. In this study, the observed predominance of STAT1-mediated Th1 polarization may be attributed to the earlier phosphorylation kinetics of STAT1 compared to STAT3 activation. This temporal advantage enables IFN-γ signaling to establish Th1 commitment before the formation of a Th17-favorable microenvironment. Moreover, activated STAT1 suppresses the IL-17 locus through epigenetic modifications, thereby attenuating RORγt-driven Th17 differentiation—a mechanism recently demonstrated in neuroinflammatory models [40]. Conversely, STAT1-deficient animals exhibit hyperactivated STAT3/Th17 pathways, confirming that intact STAT1 signaling not only constrains Th17 responses but also reinforces Th1 dominance. These mechanistic insights further justify our focus on the STAT1-driven Th1 pathway as a pivotal node in SLE pathogenesis [41]. We focused on the Th1/Th2 axis—a pathway repeatedly linked to autoantibody production, disease severity, and organ damage in SLE [42].

Notably, while Th1/Th2 imbalance is central to the immune dysregulation in SLE, the Treg/Th17 axis also plays a critical role. In patients with active disease, the frequency of Th17 cells in peripheral blood rises with worsening disease severity, while Tregs exhibit both numerical deficiency and functional impairment. This leads to a markedly reduced Treg/Th17 ratio, which undermines the suppression of autoreactive T and B cells, thereby perpetuating autoantibody production and end-organ injury. Although the present study focuses on the contribution of Th1/Th2 imbalance to SLE pathogenesis, future therapeutic approaches may benefit from simultaneously targeting the Th1/Th2/Th17/Treg axes to achieve a more robust restoration of immune tolerance in SLE patients [43, 44].

Pseudotime trajectory analysis further demonstrated that T cell exhaustion phenotypes dynamically evolved across differentiation stages and reinforced immune tolerance breakdown through LGALS9-CD45-mediated crosstalk, suggesting a self-amplifying mechanism for immune dysregulation. Th1 dominance is a recognized feature of autoimmune diseases, contributing to tissue damage via IFN-γ and macrophage activation [45]. We provide mechanistic evidence that EEF1A1 exacerbates Th1/Th2 imbalance by enhancing STAT1-dependent Th1 differentiation. In OE-eEF1A1 mice, Th1 cells were elevated in both spleen and blood, while Th2 cells declined, directly recapitulating the Th1-skewed phenotype observed in human SLE patients [46]. This imbalance likely synergizes with monocyte expansion to sustain chronic inflammation. Targeting EEF1A1 could thus restore immune equilibrium, a strategy supported by existing therapies that modulate Th1 responses [47]. By examining how EEF1A1 regulates STAT1 activity and biases Th cell differentiation, we mechanistically confirmed the contribution of ubiquitination-induced T cell dysfunction to SLE pathogenesis.

Machine learning integration of bulk RNA-seq cohorts (GSE81622 and GSE50772) prioritized EEF1A1 among 394 hub genes, with LASSO regression outperforming random forest in identifying SLE-specific drivers. Cross-cohort validation confirmed EEF1A1's diagnostic accuracy, highlighting its biomarker potential. EEF1A1's dual role as a ubiquitination-related driver and STAT1 activator positions it as a promising biomarker. Its elevated expression in SLE patient blood correlates with disease activity, suggesting utility in monitoring. Preclinically, EEF1A1 knockdown ameliorated T cell dysfunction, while its overexpression worsened renal pathology, highlighting its therapeutic potential. The in vivo demonstration of EEF1A1-driven STAT1 activation and Th1/Th2 imbalance further validates its translational relevance. Given the high heterogeneity of SLE, the clinical utility of EEF1A1 as a serum biomarker requires further validation in prospective cohorts, particularly its correlation with SLEDAI scores and lupus nephritis flares [48]. Single-cell transcriptomics has revealed a specific elevation of EEF1A1 in the peripheral blood of SLE patients, providing functional support for its potential as a serological indicator [49]. Although we have identified that EEF1A1 contributes to SLE pathogenesis via regulating the STAT1 pathway, its upstream activating mechanisms remain incompletely elucidated. Future studies should employ integrated multiomics analyses to systematically decipher the molecular network through which EEF1A1 drives immune imbalance, facilitating the development of targeted interventional strategies.

Small molecules targeting EEF1A1's ubiquitination interface or STAT1-binding domain could be explored. Notably, existing STAT1 inhibitors might be repurposed for SLE treatment, guided by our mechanistic insights [50]. While our study provides robust evidence, several questions remain unresolved. First, the molecular mechanism by which EEF1A1 activates the STAT1 signaling axis requires further elucidation. Future studies should resolve whether EEF1A1 directly recruits kinases to phosphorylate STAT1 or indirectly modulates phosphatase activity to sustain its activation. Second, therapeutic exploration must prioritize testing EEF1A1-targeted strategies—such as small-molecule inhibitors disrupting its STAT1-binding domain or ubiquitination modulators—in humanized SLE models to validate translational potential. Finally, clinical validation should expand to diverse patient cohorts to confirm EEF1A1's biomarker utility and subset-specific therapeutic relevance, particularly in patients with STAT1-driven Th1/Th2 imbalance. It should be noted that we acknowledge the limitation of the sample size in this study for a highly heterogeneous disease such as SLE, and further validation in larger cohorts is necessary.

## 5. Conclusions

This study establishes EEF1A1 as a pivotal molecular hub in SLE pathogenesis, orchestrating ubiquitination defects, STAT1-mediated Th1 polarization, and end-organ damage. Through multiomics integration and cross-species validation, we demonstrate that EEF1A1 accumulation in SLE disrupts proteasomal degradation, amplifies p-STAT1, and skews Th1/Th2 balance, culminating in renal injury. The conserved role of EEF1A1 in driving STAT1 activation across human and murine models highlights its potential as both a biomarker and therapeutic target. Future efforts should prioritize dissecting EEF1A1-STAT1 interaction mechanisms and translating these findings into targeted therapies, such as STAT1 inhibitors or EEF1A1 modulators, to restore immune homeostasis in SLE. Our work bridges molecular dysregulation, immune imbalance, and clinical manifestations, offering a roadmap for precision medicine in autoimmune diseases.

## Figures and Tables

**Table 1:** The sequences of the primers.

Gene	Forward(5′-3′)	Reverse(5′-3′)
eEF1A1	TTGCGTGAGCGGAAAGATGG	TTGCCCGAATCTACGTGTCC
STAT1	TCTGGAAAACGCCCAGAGATT	CTACTTCCTCTGTTCTGCAAGG
T-bet	GCCAAAGGATTCCGGGAGAA	CCTGGGGAACCACATCCTTC
GATA3	GCTGAATCGAAAGAGAGCAGAC	GGCATTCCTCCTCCAGAGTG
IL-4	ACCTCGACTCGCCTACAAAG	TTCCTGTCGAGCCGTTTCAG
IL-17	TAATGGCCCTGAGGAATGGC	AGGAAGCCTGAGTCTAGGGG
IFN-γ	TGCAATCTGAGCCAGTGCTT	GCACCAGGCATGAAATCTCC
GAPDH	ATGGGCAGCCGTTAGGAAAG	AGGAAAAGCATCACCCGGAG

*Note:* GAPDH served as the internal reference gene, and relative mRNA expression was calculated using the 2^−△△CT^ method.

## Data Availability

The data that support the findings of this study are available in Gene Expression Omnibus at https://www.ncbi.nlm.nih.gov/geo/query/acc.cgi, reference number GSE135779, GSE81622, and GSE50772. These data were derived from the following resources available in the public domain:   - GSE135779, https://www.ncbi.nlm.nih.gov/geo/query/acc.cgi?acc=GSE135779   - GSE81622, https://www.ncbi.nlm.nih.gov/geo/query/acc.cgi?acc=GSE81622   - GSE50772, https://www.ncbi.nlm.nih.gov/geo/query/acc.cgi?acc=GSE50772

## Nomenclature

ACR: American College of Rheumatology
Co-IP: Co-immunoprecipitation
EULAR: European League Against Rheumatism
NMF: Non-negative matrix factorization
PBMCs: Peripheral blood mononuclear cells
PCs: Principal components
RF: Random forest classifier
SDS-PAGE: Sodium dodecyl sulfate-polyacrylamide gel electrophoresis
TFs: Transcription factors
SLEDAI: Systemic lupus erythematosus disease activity index.

## Supporting Information

Supporting Information Figures S1 provides the validation of EEF1A1 ubiquitination status and constructed plasmids, Figure S2 provides the analysis of EEF1A1 and STAT1 mRNA expression and Th1/Th2 cell proportions, and Figure S3 evaluates the mRNA expression of Th cell-related cytokines and transcription factors upon EEF1A1 knockdown and overexpression. Table S1 provides the baseline data of SLE patients and normal controls, and Table S2 provides the quantitative histological scoring of renal pathology in mouse models.
