# Supplementary material for: Single-Cell Transcriptomics Uncover EEF1A1-Driven Ubiquitination Dysregulation in T Cell Exhaustion and SLE Pathogenesis via STAT1-Mediated Th1/Th2 Imbalance
Source: Mediators Inflamm. 2025 Nov 11;2025:3708640. doi: 10.1155/mi/3708640 (PMC12626703; doi:10.1155/mi/3708640)
Supplement: Supporting Information — Figures S1 provides the validation of EEF1A1 ubiquitination status and constructed plasmids, Figure S2 provides the analysis of EEF1A1 and STAT1 mRNA expression and Th1/Th2 cell proportions, and Figure S3 evaluates the mRNA expression of Th cell-related cytokines and transcription factors upon EEF1A1 knockdown and overexpression. Table S1 provides the baseline data of SLE patients and normal controls, and Table S2 provides the quantitative histological scoring of renal pathology in mouse models. [file 3708640.f1.zip › supplementary Table 2.docx]

**Supplementary Table 2. HE staining score of mouse kidney tissue**

| Group | Control | | | SLE+OE-NC | | | SLE+OE-eEF1A1 | | |
| --- | --- | --- | --- | --- | --- | --- | --- | --- | --- |
| Number | 1 | 2 | 3 | 1 | 2 | 3 | 1 | 2 | 3 |
| Cell proliferation within the glomerulus | 1 | 0 | 0 | 2 | 1 | 2 | 3 | 2 | 3 |
| Capillary hyperplasia | 0 | 1 | 1 | 3 | 2 | 3 | 3 | 3 | 2 |
| Mesangial hyperplasia | 0 | 0 | 0 | 1 | 1 | 2 | 2 | 3 | 2 |
| Glomerulosclerosis | 0 | 1 | 0 | 3 | 2 | 1 | 2 | 3 | 2 |
| Renal interstitial fibrosis | 0 | 0 | 1 | 1 | 2 | 1 | 2 | 2 | 3 |
| Interstitial inflammation | 0 | 0 | 0 | 1 | 0 | 1 | 2 | 2 | 1 |
| Cell infiltration | 0 | 0 | 0 | 2 | 2 | 1 | 3 | 2 | 3 |
| Total | 1 | 2 | 2 | 13 | 10 | 11 | 17 | 17 | 16 |
